# Ultrafast coherent exciton dynamics in size-controlled perylene bisimide aggregates

**DOI:** 10.1063/1.5124148

**Published:** 2019-11-26

**Authors:** Seongsoo Kang, Christina Kaufmann, Yongseok Hong, Woojae Kim, Agnieszka Nowak-Król, Frank Würthner, Dongho Kim

**Affiliations:** 1 Spectroscopy Laboratory for Functional π-Electronic Systems and Department of Chemistry, Yonsei University, Seoul 03722, South Korea; 2 Institut für Organische Chemie and Center for Nanosystems Chemistry, Universität Würzburg, Am Hubland, 97074 Würzburg, Germany

## Abstract

For H-aggregates of perylene bisimide (PBI), it has been reported that upon photoexcitation, an initially delocalized Frenkel exciton is localized by excimer formation. However, in recent studies, the beforehand exciton dynamics preceding the excimer formation was suggested in larger aggregates consisting of at least more than 10-PBI subunits, which was not observed in small aggregates comprising less than four-PBI subunits. This feature implies that the size of molecular aggregates plays a crucial role in the initial exciton dynamics. In this regard, we have tried to unveil the initial exciton dynamics in PBI H-aggregates by tracking down the transient reorientations of electronic transition dipoles formed by interactions between the PBI subunits in systematically size-controlled PBI H-aggregates. The ultrafast coherent exciton dynamics depending on the molecular aggregate sizes can be distinguished using polarization-dependent femtosecond-transient absorption anisotropy spectroscopic measurements with a time resolution of ∼40 fs. The ultrafast decay profiles of the anisotropy values are unaffected by vibrational relaxation and rotational diffusion processes; hence, the coherent exciton dynamics of the PBI H-aggregates prior to the excimer formation can be directly revealed as the energy migration processes along the PBI H-aggregates.

## I. INTRODUCTION

Supramolecular dye assemblies are drawing considerable attention among numerous research groups owing to the possibilities of promising applications in the fields of photonics, optoelectronics, and organic photovoltaics.^1–4^ In particular, long-lived Frenkel excitons in H-aggregates forming a cofacile organization play an important role in the efficient energy transfer preceding energy loss through exciton relaxation processes.^5,6^ Among several representative molecular entities in supramolecular dye assemblies available as a medium for the efficient energy transfer, perylene bisimide (PBI) dyes have been considered as suitable candidates for artificial light-harvesting applications since PBI dyes show a strong thermodynamic driving force for π–π stacking into one-dimensional H-aggregates by monomeric π-π stacks as well as remarkable photostability and high fluorescence quantum yields.^7–10^ However, it has been challenging to reveal the fundamental exciton dynamics because of the formation of a low energy trapping state in PBI aggregates, which was commonly called as the excimer state, exciton localized state. Many studies reported that the localization processes of the initially generated exciton occur on an ultrafast time scale competing with the energy transfer processes.^11–15^ Accordingly, spectroscopic investigation on an ultrafast time scale prior to excimer formation can provide further insights into the fate of primarily generated excitons. Furthermore, understanding the ultrafast exciton dynamics in molecular H-aggregates is also highly important for advances in material performance.

Recently, using femtosecond broadband upconversion spectroscopy (FLUPS) experiments, we reported that initially photoinduced Frenkel excitons show spectral evolutions from the initial Frenkel exciton state to the excimer state within 200 fs. This process was monitored regardless of the length of the PBI H-aggregates.^9^ However, notably, only larger aggregates consisting of infinite PBI subunits showed additional exciton dynamics preceding the excimer formation with a time constant of sub-80 fs, which was suggested as the exciton migration along the PBI stacks. Although this suggestion provided crucial information of the Frenkel exciton dynamics, an intrinsically limited time-resolution (∼110 fs) of FLUPS experiments constrained a clear observation of the ultrafast Frenkel exciton dynamics. Therefore, the comparative analysis of length-dependent exciton dynamics in the PBI H-aggregates stimulates the design of more accurate measurements for the observation of the efficient energy transfer processes by using a higher time resolution compared to FLUPS measurements.^5,6^ With this objective, we devised and performed time-resolved polarization-dependent spectroscopic studies, which are expected to provide valuable information about the ultrafast dynamics of the Frenkel exciton related to the depolarization channel in the PBI molecular aggregates. For this analysis, structural information of aggregates is essential because the excimer formation processes are largely affected by intermolecular angles and distances between the neighboring PBIs depending on the types of PBI core substituents.^16–18^ In addition, the extent of mixing between charge transfer (CT) and Frenkel exciton states can affect the localization processes prior to the excimer state formation depending on the stacked structures.^19,20^ In this context, we prepared the following series of size-controlled PBI H-aggregates. The triphenyl spacer unit in PBI **1** directs a folding of the molecule into a dimer stack, while the spacer unit in PBI **2** is optimized to direct the self-assembly of two molecules into a bimolecular complex bearing a tetramer stack.^15,16^ Finally, PBI **3** molecules form extended oligomer stacks by self-assembly of the monomers.^9^ In the following, we will call these systems dimer, tetramer, and extended oligomeric aggregates. Notably, as will be discussed later, these PBI stacks exhibit somewhat different geometries in terms of rotational displacements and distances between the neighboring PBI subunits. The goal of the current study is now to obtain further insight into the ultrafast localization processes of Frenkel excitons.^9,15,16^ With a short pulse of ∼40 fs from our noncollinear optical parametric amplifier (NOPA) system (Fig. S2), we performed femtosecond transient absorption (fs-TA) experiments to confirm the exciton localization processes depending on the sizes and structures of the PBI H-aggregates. Moreover, based on the analysis of fs-TA spectroscopic measurements, polarization-dependent spectroscopic studies, fs-transient absorption anisotropy (TAA), were performed to unveil the excitonic interactions by tracking down the transient orientation of transition dipoles in helically π–π stacked structures of PBI H-aggregates.

## II. RESULTS AND DISCUSSION

### A. Steady-state absorption and fluorescence

**FIG. 1.**
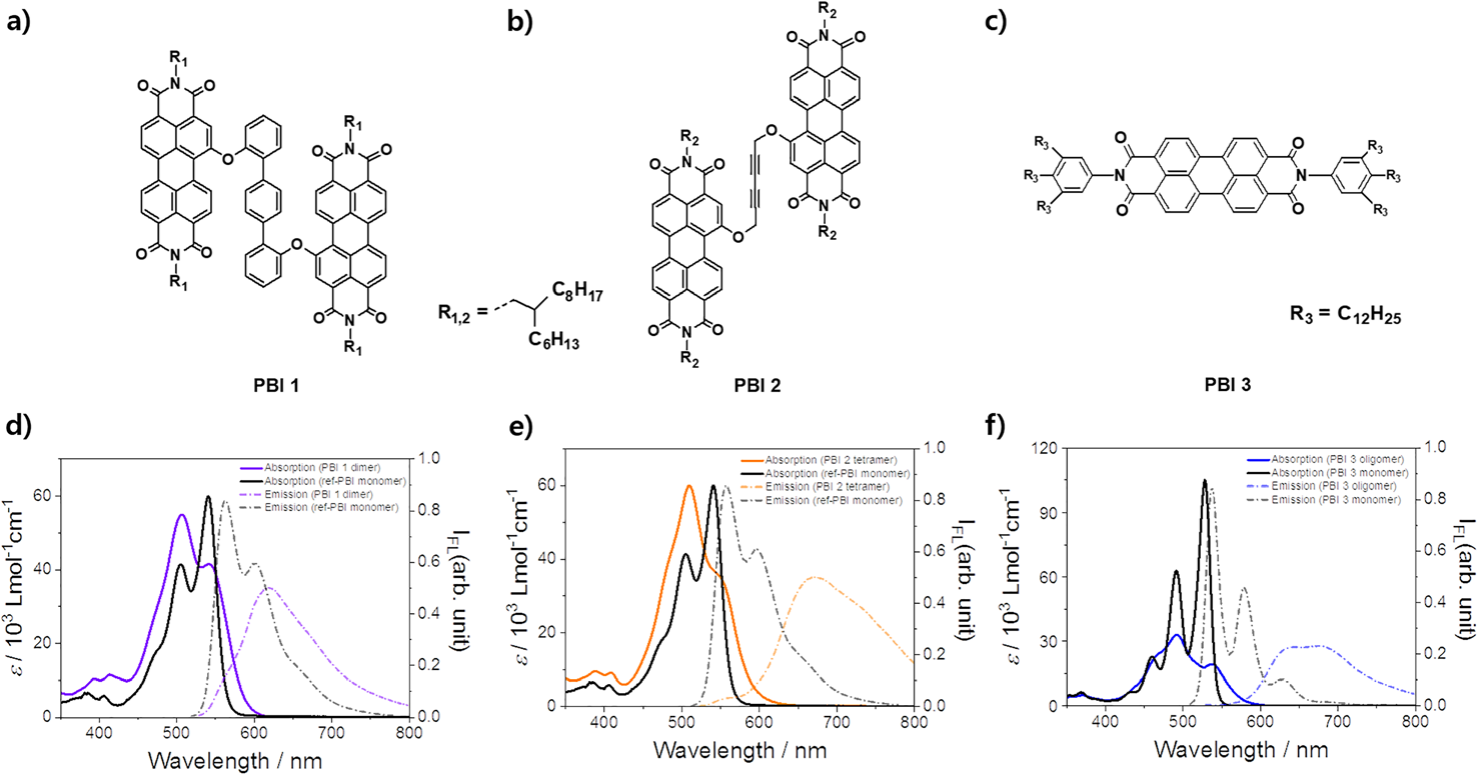
Molecular structure (a) of PBI **1** (left), (b) PBI **2** (middle), and (c) PBI **3** (right). (d) Colored steady-state absorption and emission spectra of concentrated PBI **1** (3.0 × 10^−4^ M, folded “dimer” stack) in toluene and the diluted ref-PBI monomer in chloroform. (e) Those of concentrated PBI **2** (3.0 × 10^−4^ M, self-assembled bimolecular complex, i.e., tetramer stack) in tetrahydrofuran and the diluted ref-PBI monomer in chloroform. (f) Those of concentrated PBI **3** (1.0 × 10^−3^ M, extended oligomer stack) in methylcyclohexane and the diluted PBI **3** monomer in chloroform.

First, steady-state measurements of PBIs **1**, **2**, and **3** were performed in toluene, tetrahydrofuran (THF), and methylcyclohexane (MCH) at room temperature, respectively, to explore the optical properties of the PBI H-aggregates in the ground state (Fig. 1). PBIs **1**, **2**, and **3** form exclusively dimeric, tetrameric, and extended oligomeric PBI stacks under certain conditions.^9,15,16^ Although bay-substituted PBIs show different molecular characteristics from non-bay-substituted PBIs, in this report, we do not consider this issue to clarify differences in exciton dynamics due to the size of PBI H-aggregates. Under low concentrated conditions (i.e., below 1.0 × 10^−7^ M) in a good solvating solvent (i.e., CHCl_3_), the absorption spectra of both the ref-PBI monomer (bay-substitution) and the PBI **3** monomer (non-bay-substitution) show a vibrational progression over the S_0_–S_1_ electronic transition coupled to the symmetric C-C vinyl stretching modes of the PBI core with approximately 1400 cm^−1^ (Fig. S1).^17,18^ In contrast, when PBI aggregates are formed in highly concentrated solutions in a poorly solvating solvent, the absorption spectra of a series of PBI aggregates exhibit a hypsochromic shift and broad spectral features resulting from the electronic interactions between the transition dipoles along the long axis of the π-stacked monomer.^18^ According to the electronic interactions between the PBI subunits, the H-type aggregates induce positive excitonic coupling and energetic splitting of an excitonic state, giving rise to the formation of the allowed upper and forbidden lower energy states.^18,21–24^ In this regard, the reversed intensities of the 0–0 and 0–1 bands in the absorption spectrum of the PBI aggregates well reflect the excitonic characteristics of the H-aggregates.^13^ Moreover, in the PBI aggregates, the steady-state emission spectra reveal far red-shifted and broad features, indicating typical excimer fluorescence as previously reported.^9,20^ In contrast, all the emission spectra of the PBI monomers show a mirror image to the absorption spectra, indicating a well-resolved vibronic emission spectrum. Although the PBI H-aggregates clearly exhibit different spectral features in the steady-state absorption and emission spectra compared to the monomer PBI, a disparity according to the aggregate length-dependence could hardly be distinguished by the steady-state spectral features except for the intensity ratio values of the A_0–0_ and A_0–1_ bands (A_0–0_/A_0–1_) in the absorption spectra. The ratio values show the lower intensity ratio of the A_0–0_ and A_0–1_ bands in longer PBI aggregates with the ratio values of 0.76, 0.66, and 0.53 in PBIs **1** dimer, **2** tetramer, and **3** oligomer stacks, respectively.^13,17^

### B. Computational optimization calculations

**FIG. 2.**
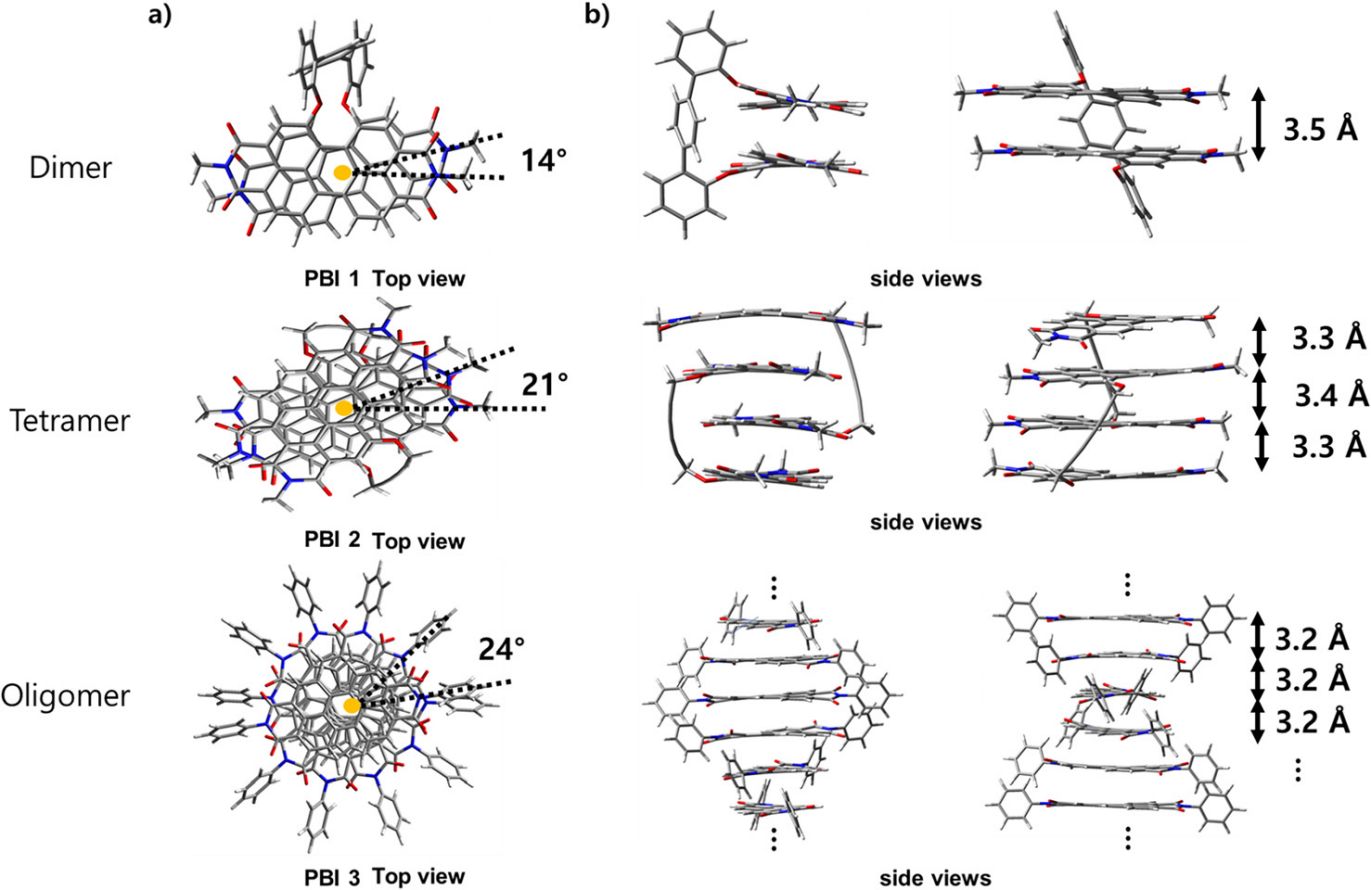
(a) Top and (b) side views of the H-type PBI aggregates (top: PBI **1** folded into a dimer stack; middle: PBI **2** self-assembled into a tetramer stack; and bottom: PBI **3** self-assembled into an extended oligomer) using the geometric optimization calculation (B97D3/def2-SVP). In the case of PBI **3** self-assemblies, only 6 subunits are used to diminish computational costs. Hydrogens are omitted, and N-substituents are changed to the alkyl group for clarity in all three aggregates.

Additionally, the density functional theory (DFT) calculations (B97D3/def2-SVP) for the geometric optimization of PBIs **1** dimer and **2** tetramer and the molecular modeling studies of the PBI **3** oligomer reveal that the three PBI aggregates form a similar intermolecular geometry.^9,15,16^ As shown in Fig. 2, the helically π-stacked structures of our PBI H-aggregates are envisioned with the intermolecular angles of 14° for PBI **1** dimer, 21° for PBI **2** tetramer, and 24° for PBI **3** oligomer with distances of 3.2–3.5 Å between the neighboring PBI subunits, respectively. All three assemblies with similar exciton coupling strength in the intermediate regime of approximately 500–900 cm^−1^ allow us to perform a comparative analysis about the Frenkel exciton dynamics in the size-controlled PBI aggregates.^15,16^

### C. Femtosecond transient absorption

**FIG. 3.**
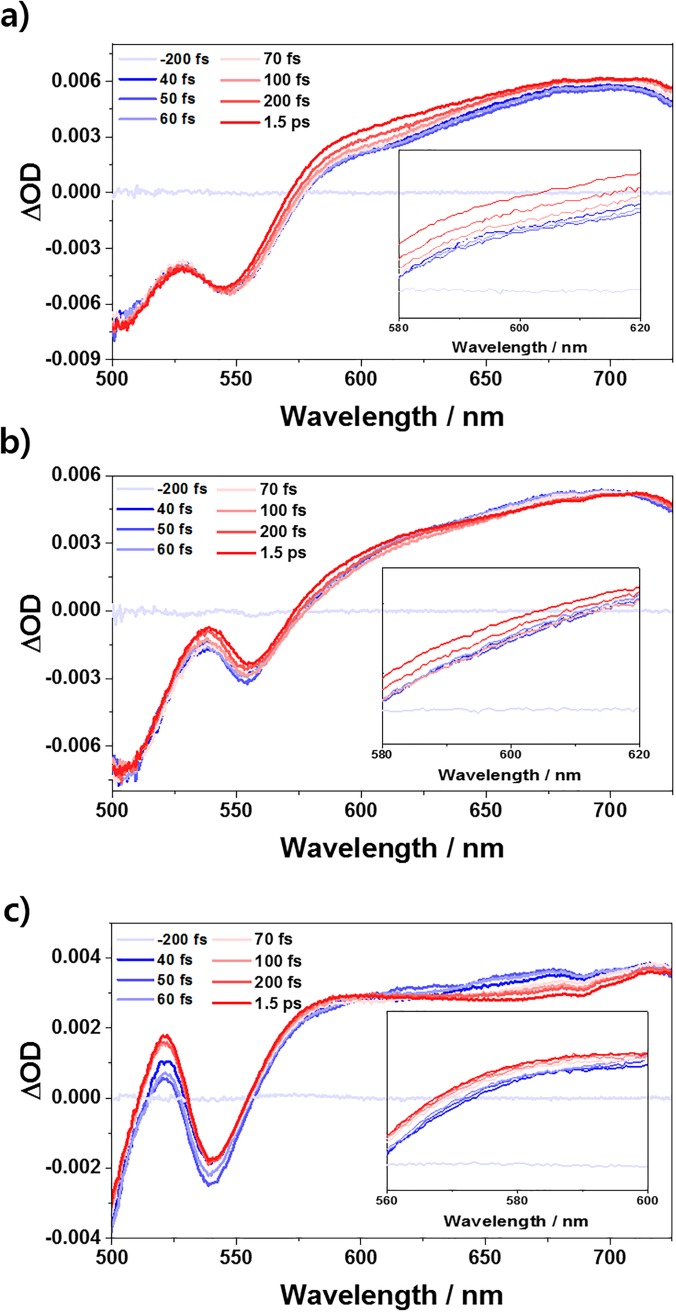
Magic angle fs-TA spectra of (a) PBI **1** (c_0_ = 0.3 mM), (b) PBI **2** (c_0_ = 0.3 mM), and (c) PBI **3** (c_0_ = 1.0 mM) observed in toluene, THF, and MCH, respectively, within a 1.5 ps time scale. Inset: magic angle fs-TA spectra of 580–620 nm (a) and (b) and 560–600 nm (c).

Based on studies on the ground state of the PBI H-aggregates, fs-TA measurements are performed on an ultrafast time scale to investigate the exciton dynamics of a series of PBI H-aggregates in the excited state. The excitation beam is a 530 nm-centered NOPA pulse with a time duration of sub-40 fs in all three PBI aggregates. As a reference, the fs-TA spectra of the ref-PBI monomer showed well-resolved ground state bleaching (GSB) and stimulated emission (SE) bands below 630 nm and considerably sharp excited state absorption (ESA) signals peaking at 700 nm, indicating no distinct ultrafast dynamics.^11,25–27^ However, in all the PBI aggregates, the TA spectra [Figs. 3(a)–3(c)] in the visible wavelength region exhibit similarly broad GSB signals below 570 nm. Moreover, the positive and broad ESA signals from 570 nm onward provide us with a definite evidence of the S_1_ → S_n_ transition resulting from the excimer formation in the excited state.^9,11,15^ The rise components of the ESA bands from the approximately 560–620 nm region (Fig. 3, insets) in all the PBI aggregates are noteworthy. The ultrafast components imply the disappearance of the vibronic emission spectra, given that the emission spectra by the Frenkel exciton state are overlapped with the SE bands of PBI **1**, **2**, and **3** aggregates (Fig. 3, insets), respectively. In this region, fs-TA rise profiles (Fig. S3) in PBI **1** and **2** H-aggregates show similar time constants within a few hundreds of femtoseconds as monitored in the previous works by Sung *et al.*^9,15^ It can be considered as a stimulated emission quenching process of the Frenkel exciton due to excimer formation. Therefore, we suggest that the rise signals of the ESA band of a series of PBI H-aggregates are induced by the localization processes of the Frenkel exciton on an ultrafast time scale. However, the presence of complex states resulting from the coupling interactions between the PBI monomers exhibits broader and less clear structures of the fs-TA spectra in the PBI **3** oligomer compared to those of the PBI **1** dimer and PBI **2** tetramer, which does not facilitate a further analysis of the spectra obtained from the polarization-independent fs-TA (magic angle).^11,13^ Indeed, band integrals^28,29^ *I*(690, 710) for purely tracking down the excimer formation processes not to be interrupted from the GSB and SE signals show hardly distinctive decays in all three PBI aggregates (Fig. S4),

$$I\left(\lambda_{1},\lambda_{2}\right)=\frac{1}{\ln\left(\lambda_{2}∕\lambda_{1}\right)}\int_{\lambda_{1}}^{\lambda_{2}}\Delta A\left(t,\lambda \right)d\lambda ∕\lambda .$$ (1)

In our previous FLUPS analysis based on Spano's theoretical model, the extent of exciton delocalization in the cofacial PBI aggregates was reproduced by the 0–0 to 0–1 peak ratio in the time-resolved fluorescence spectra to distinguish the difference in ultrafast excimer formation processes in a series of PBI H-aggregates,^9,15^

$$N_{\mathrm{coh}}\left(t^{*}\right)=\lambda^{2}\frac{I^{0-0}\left(t^{*}\right)}{I^{0-1}\left(t^{*}\right)}.$$ (2)

From this analysis, the exciton delocalization extent in the H-type PBI aggregates was represented by the values of the coherence length (*N_coh_*), whose ultrafast decay profiles correspond to the localization processes of a delocalized exciton. We confirmed that the dimer and the tetramer have a delocalized Frenkel exciton with the initial *N*_coh_ values of 2 and ∼3 after photoexcitation, respectively, implying the generation of a fully delocalized exciton in both dimer and tetramer stacks. Afterwards, the initial *N*_coh_ decayed with the single-exponential decay time constant of 200 fs, which is related to the localization processes caused by the excimer formation of the Frenkel exciton. Meanwhile, in the PBI **3** oligomer, the transient changes in the initial *N*_coh_ (at least 3) were observed as two decay components with the time constants of sub-80 and 250 fs (double-exponential decay). The faster decay was suggested as an exciton migration of the Frenkel exciton along the PBI **3** oligomer, which reflects the localization of the partially delocalized exciton, because any changes of the 0–1 to 0–v′ (v′ > 1) peak ratio in the time-resolved fluorescence spectra, indicating excimer formation processes, were not observed up to approximately 110 fs. However, the intrinsic time-resolution limit of the FLUPS experiments (∼110 fs) restricted obtaining direct information of the ultrafast Frenkel exciton dynamics.

### D. Femtosecond transient absorption anisotropy

**SCHEME 1.**
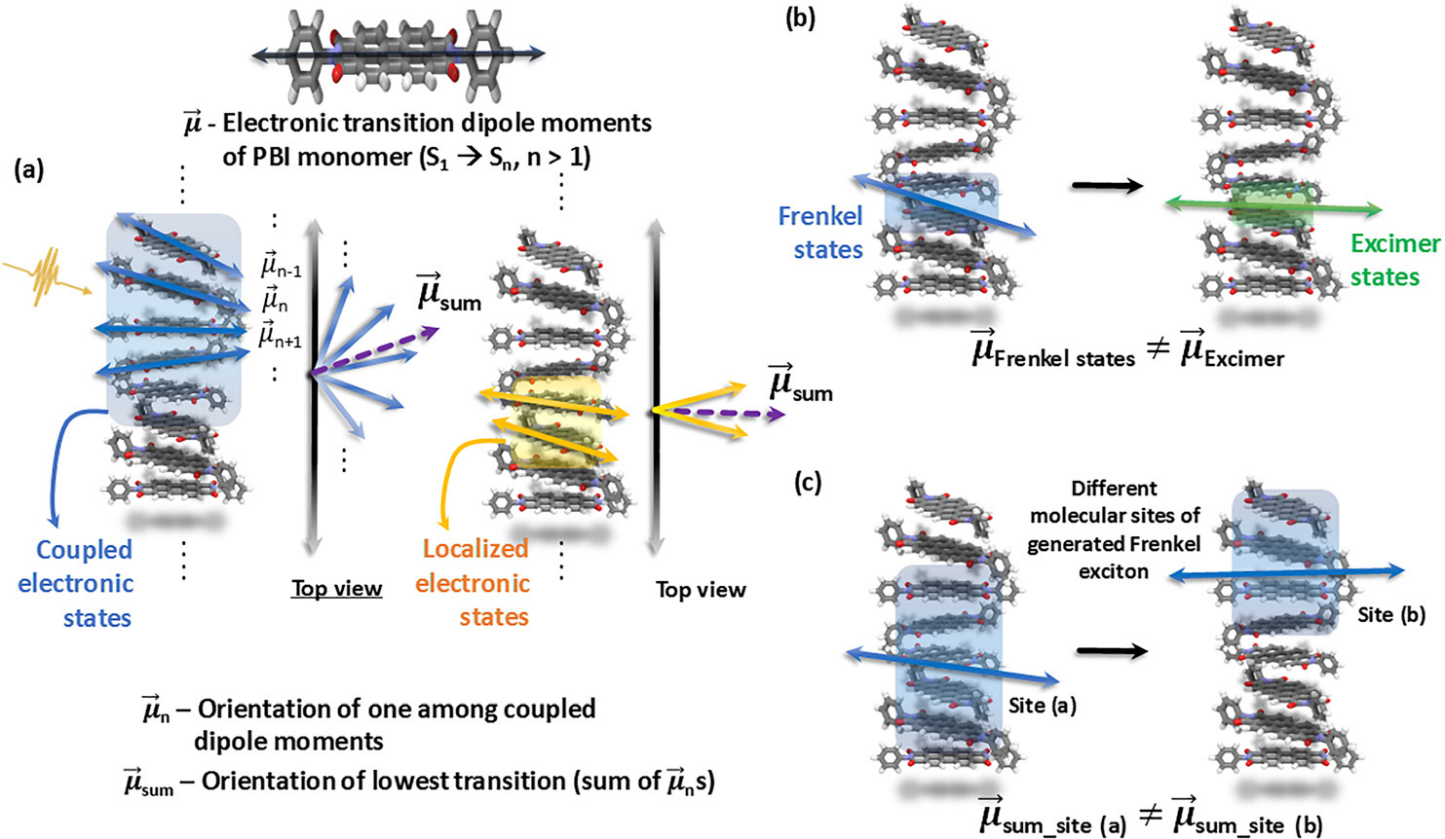
Representation of our three assumptions; (a) orientational changes of the coupled transition dipoles according to the extent of localization of the Frenkel exciton in molecular aggregates (colored block: extent of exciton delocalization); (b) different orientations in Frenkel and excimer states in the case of similar numbers of coupled transition dipoles; and (c) different orientations according to the molecular sites generated by the Frenkel exciton after photoexcitation. These three assumptions come from the information that the arrangement of the PBI H-aggregates is stacked with rotational displacements.

**FIG. 4.**
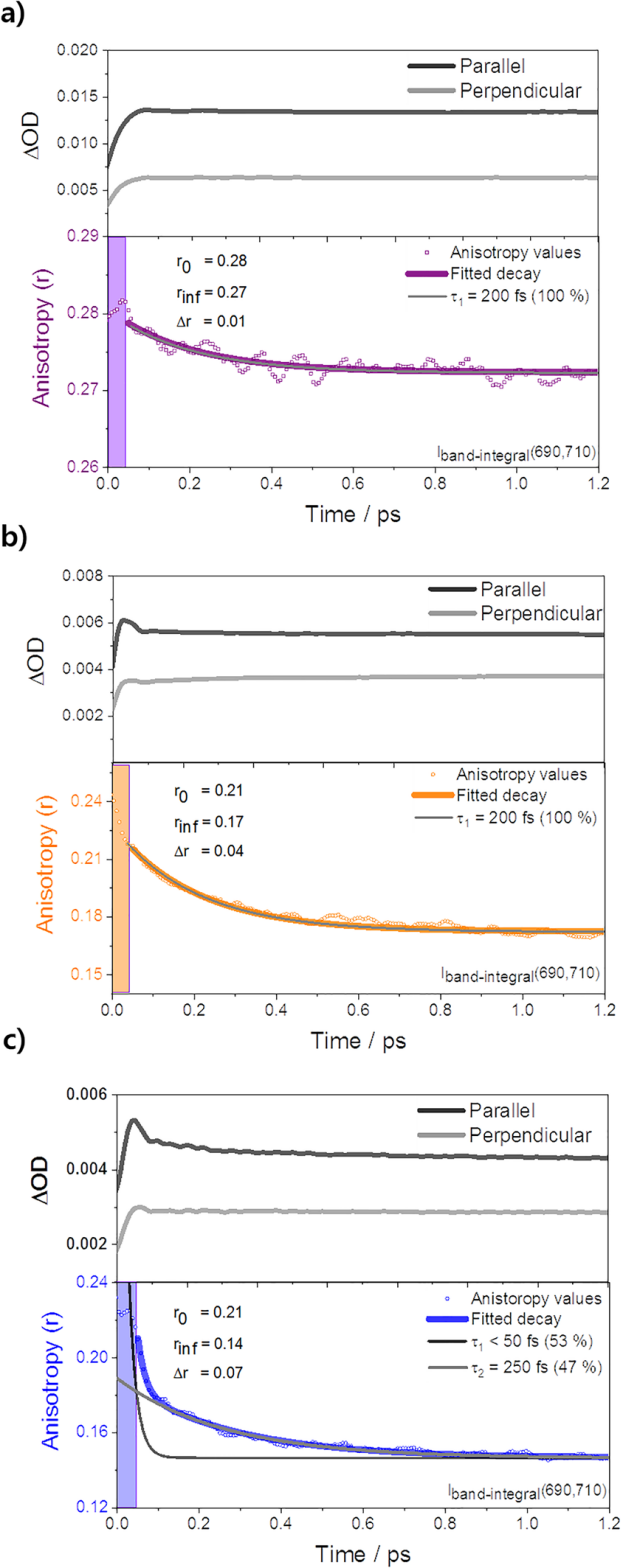
Each upper graph indicates the fs-TA decay profiles exhibiting the relative polarization direction between the pump and the probe laser pulses (black: parallel; gray: perpendicular). Each lower graph indicates the anisotropic decay profiles: (a) PBI **1** dimer (purple), (b) PBI **2** tetramer (orange), and (c) PBI **3** extended oligomer (blue) in toluene, THF, and MCH, respectively. Note that PBIs **1** and **2** show a monoexponential one, while PBI **3** shows double-exponential anisotropy decay profiles. Inset: values of the initial anisotropy (r_0_) and the final anisotropy (r_inf_) after 1.2 ps and the difference (Δr) between r_o_ and r_inf_. All values are probed at band integrals*I*(690, 710).

In this context, we proceed with the polarization-dependent experiments using short pulses of ∼40 fs to directly explore the ultrafast coherent dynamics of the Frenkel exciton based on the fs-TA spectra and FLUPS measurements of size-controlled PBI H-aggregates. Three assumptions can be envisioned and preconditioned prior to an analysis for the measurements (Scheme 1). Considering the rotational displacements of the PBI H-aggregates, first, the localization processes of the Frenkel exciton lead to the reduction in the number of coupled PBI monomers, which influences the orientations of a vector sum of the transition dipoles after photoexcitation.^9,15^ Second, the difference between a delocalized Frenkel exciton and the excimer state can induce the orientation changes of coherent transition dipoles (i.e., mixing of the Frenkel and CT states in the process of excimer formation).^19,20,25^ Finally, considering the conformational heterogeneity of the molecular aggregates in the solution, the molecular sites of a photoexcited Frenkel exciton in the aggregates can affect the orientation of the vector sums of the transition dipoles by altering the extent of electronic coupling between the PBI subunits.^11^ Consequently, we suggest that the polarization-dependent spectroscopic studies can unveil the ultrafast coherent dynamics of the Frenkel exciton like exciton migration and excimer formation according to the anisotropic changes in a series of size-controlled PBI aggregates. The anisotropy values of each PBI aggregate were obtained from the fs-TAA experiments by measuring each decay profile of the two fs-TA spectra with polarized pump pulses of parallel and perpendicular directions relative to the axis of the probe pulses (upper graphs in Fig. 4).^11,19^ Under the same conditions as fs-TA experiments, the depolarizing evolution of the anisotropy values was obtained by band integrals *I*(690, 710) analysis in all the PBI aggregates.

In the case of the ref-PBI monomer, the ultrafast depolarization process was not observed in the entire wavelength range (Fig. S5), showing the constant value of anisotropy (r = 0.4). This result implies that the orientations of the transition dipoles for S_0_ → S_1_ and S_1_ → S_n_ (n, higher state) are nearly the same as the direction of the induced excitation light in the time window of 80 ps. However, the ultrafast depolarization is clearly demonstrated in a series of PBI aggregates. As mentioned previously, the ESA regions limited for band integral *I*(690, 710) analysis allow us to selectively monitor the localization process of delocalized Frenkel excitons (lower graphs in Fig. 4). Based on the calculated molecular structures in the ground state (Fig. 2) and reported initial *N*_coh_ values of PBI aggregates, we can estimate the fs-TA anisotropy values as modeled to obtain fluorescence anisotropy values (supplementary material). However, we can observe anisotropy values from approximately 60 fs considering the time-resolution of our fs-TA apparatus. Accordingly, the initial anisotropy value (r_0_) is selected at approximately 60 fs after photoexcitation. Particularly, it must be noted that the CT state can be considered as an intermediate or virtual state for the formation of an excimer state; hence, the initial r_0_ values less than expected (lower insets in Fig. 4) could be corresponding to the Frenkel state perturbed by being mixed with the CT state.^19,20,25^ In the PBI **1** dimer, the initial anisotropy value compared to those of the PBI **2** tetramer and PBI **3** oligomer shows a rather high r_0_ value because the nonparallel excitonic interaction of only the two transition dipoles contributes to canceling out the absorbing dipole vectors, even considering the mixed state with the CT state. It is also consistent with a small deviation (approximately 14°) of the intermolecular stacking angle [Fig. 2(a)].^13^ Meanwhile, the initial anisotropic values in both the PBI **2** tetramer and the PBI **3** oligomer show similarly low r_0_ values of approximately 0.21. It may arise from a large offset by the interaction of at least three transition dipoles with the rotational displacement of the coupled transition dipoles according to the initial molecular sites of the generated Frenkel excitons. Meanwhile, in addition to canceling out between coupled transition dipoles, especially in the PBI **2** tetramer, the solvent polarity of THF can affect low r_0_ values since the mixed states of the Frenkel state and the CT state may include more CT characteristics compared to those of the PBI **3** oligomer in nonpolar MCH.^30^

As mentioned above, the mixed states can influence the decrease in the initially observed r_0_ values. However, given the orientation of the dipole moments caused by the CT state oriented along the aggregate scaffold,^20^ the orientation changes of the vector sum according to the number of coherently coupled transition dipoles between PBI subunits are hardly affected by those of the CT state. Orientations of the CT state dipoles and the Frenkel exciton state dipoles are perpendicular to each other. In this regard, we suggest that the ultrafast depolarization processes in PBI H-aggregates can be monitored independent of the CT-perturbed state. Therefore, the nascent dynamics of the Frenkel exciton can be selectively monitored by the TAA decay profiles.

**SCHEME 2.**
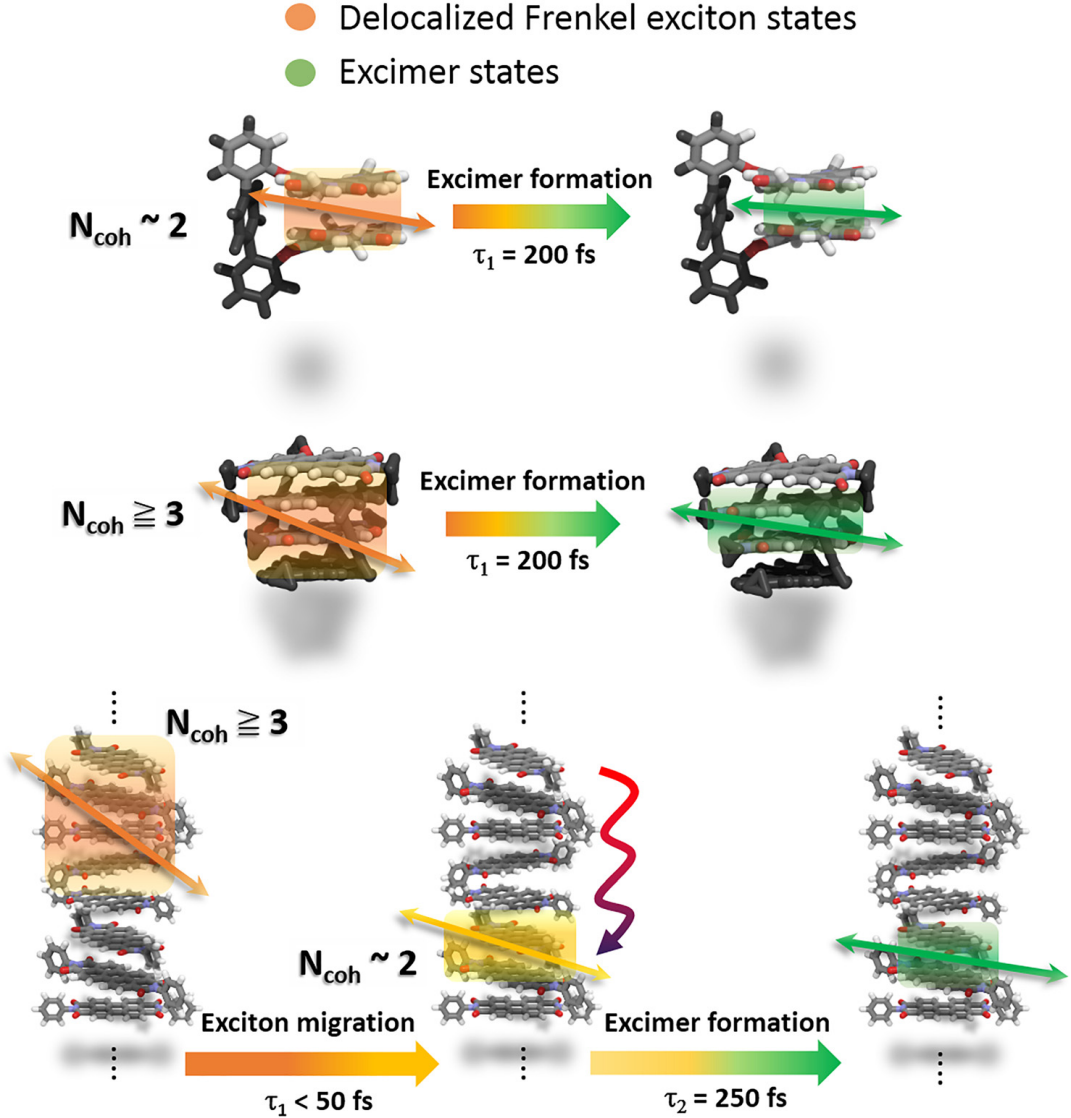
Illustration of the length-dependent localization processes of a delocalized Frenkel exciton in H-type PBI aggregates on an ultrafast time scale. (top: PBI **1** dimer; middle: PBI **2** tetramer; and bottom: PBI **3** oligomer).

For a qualitative analysis of the ultrafast depolarization processes in a series of size-controlled PBI aggregates, the final anisotropy value (r_inf_) was chosen on a time scale of 1.2 ps to minimize the disturbances of the value by the relaxation processes of the excimer with a time constant of a few picoseconds.^9,15^ In the PBI **1** dimer, the TAA decay profiles indicate minor depolarization (Δr = 0.01) processes compared to those of the PBI **2** tetramer and PBI **3** oligomer, implying that the nature of the Frenkel exciton and that of the excimer state are different inducing the orientation changes of the transition dipoles within 200 fs [Scheme 1(b)]. In contrast, the depolarization dynamics in the PBI **2** tetramer and the PBI **3** oligomer exhibits sharper anisotropy decay profiles than that of the PBI **1** dimer. While the decay profiles of the anisotropy values in both the PBI **1** dimer and the PBI **2** tetramer are fitted to the monoexponential decay with a time constant of 200 fs, large depolarization (Δr = 0.04) processes in the PBI **2** tetramer particularly reflect that the extent of canceling out of the coupled transition dipoles with four monomeric moieties is more significant. It corresponds to a notable reduction in the initial coherent length of at least three as observed in previous FLUPS studies.^9,15^ Meanwhile, the largest depolarization dynamics (Δr = 0.07) of the PBI **3** oligomer is fitted with double-exponential components having time constants of sub-50 fs and 250 fs [lower in Fig. 4(c)]. This result was obtained from the condition without the laser-pump-power-dependency to avoid the contamination of the TAA decay profiles by exciton-exciton annihilation (EEA) processes in molecular aggregates.^11^ Primarily, the faster decay is devoid of the EEA processes, considering that the EEA processes in the PBI **3** oligomer were monitored on the time scale of approximately 1 ps. Despite a small deviation of r_0_ in both the PBI **2** tetramer and the PBI **3** oligomer caused by the similar *N*_coh_ values of approximately 3, the depolarization processes exhibit the larger and faster reduction of the anisotropy values in the PBI **3** oligomer than the PBI 2 tetramer. Furthermore, the initial decay profile is fitted with slightly larger depolarization amplitudes (53%) than the latter one (47%). Hence, we can directly propose that the exciton migration along the columnar stacks of the PBI **3** oligomer accelerates the additional decoherence of the coupled transition dipoles. Afterwards, the latter decay (250 fs) shows similar depolarization processes due to the excimer formation like those in the PBI **1** dimer. This result implies that the exciton migration along the PBI molecular stacks sharply localizes a delocalized Frenkel exciton reducing the size of the excitonic wavefunction.^9,15^ An almost localized Frenkel exciton then forms excimer, indicating a complete localization within 250 fs (Scheme 2).^9,15,31,32^

## III. CONCLUSIONS

As mentioned earlier, intrinsic limitations exist to clarify the ultrafast Frenkel exciton dynamics in the PBI H-aggregates using fs-TA and FLUPS studies. In this context, using short pulses of ∼40 fs, we suggested three assumptions to perform a polarization-dependent TAA spectroscopic experiment in a series of size-controlled PBI H-aggregates. The comparative ultrafast behaviors of the Frenkel exciton were confirmed with the three assumptions. We observed that the exciton migration occurred before the formation of the exciton self-trapped states (excimer) in extended oligomer aggregates missing in small aggregates (dimer and tetramer). Furthermore, this comparative study on the size-controlled PBI aggregates confirmed the coherent energy transfer. Therefore, our findings of coherent exciton coupling dynamics depending on the length of the PBI H-aggregates not only can provide a doorway to elucidate the exciton behaviors of more complicated molecular aggregates but also suggest the advances of molecular optoelectronic materials through the efficient energy transfer preceding trap states of excitons in larger aggregates.

## SUPPLEMENTARY MATERIAL

See the supplementary material for experimental details, noncollinear optical parametric amplifier (NOPA) spectrum of the pump pulse for fs-TA and fs-TAA measurements, structure, steady-state absorption, emission, and transient absorption anisotropy information on the reference monomer, transient absorption decay profiles of PBI **1** and **2**, transient absorption band integral decay profiles, and computational calculation results of PBI **1**, **2**, and **3**.
